# Effect of therapeutic interchange on medication reconciliation during hospitalization and upon discharge in a geriatric population

**DOI:** 10.1371/journal.pone.0186075

**Published:** 2017-10-19

**Authors:** Jessica S. Wang, Robert L. Fogerty, Leora I. Horwitz

**Affiliations:** 1 Department of Internal Medicine, University of California, San Francisco, San Francisco, CA, United States of America; 2 Section of General Internal Medicine, Department of Internal Medicine, Yale School of Medicine, New Haven, CT, United States of America; 3 Division of Healthcare Delivery Science, Department of Population Health, NYU School of Medicine, New York, NY, United States of America; 4 Center for Healthcare Innovation and Delivery Science, NYU Langone Health, New York, NY, United States of America; 5 Division of General Internal Medicine and Clinical Innovation, Department of Medicine, NYU School of Medicine, New York, NY, United States of America; Providence VA Medical Center, UNITED STATES

## Abstract

**Background:**

Therapeutic interchange of a same class medication for an outpatient medication is a widespread practice during hospitalization in response to limited hospital formularies. However, therapeutic interchange may increase risk of medication errors. The objective was to characterize the prevalence and safety of therapeutic interchange.

**Methods and findings:**

Secondary analysis of a transitions of care study. We included patients over age 64 admitted to a tertiary care hospital between 2009–2010 with heart failure, pneumonia, or acute coronary syndrome who were taking a medication in any of six commonly-interchanged classes on admission: proton pump inhibitors (PPIs), histamine H_2_-receptor antagonists (H2 blockers), hydroxymethylglutaryl CoA reductase inhibitors (statins), angiotensin-converting enzyme (ACE) inhibitors, angiotensin receptor blockers (ARBs), and inhaled corticosteroids (ICS). There was limited electronic medication reconciliation support available. Main measures were presence and accuracy of therapeutic interchange during hospitalization, and rate of medication reconciliation errors on discharge. We examined charts of 303 patients taking 555 medications at time of admission in the six medication classes of interest. A total of 244 (44.0%) of medications were therapeutically interchanged to an approved formulary drug at admission, affecting 64% of the study patients. Among the therapeutically interchanged drugs, we identified 78 (32.0%) suspected medication conversion errors. The discharge medication reconciliation error rate was 11.5% among the 244 therapeutically interchanged medications, compared with 4.2% among the 311 unchanged medications (relative risk [RR] 2.75, 95% confidence interval [CI] 1.45–5.19).

**Conclusions:**

Therapeutic interchange was prevalent among hospitalized patients in this study and elevates the risk for potential medication errors during and after hospitalization. Improved electronic systems for managing therapeutic interchange and medication reconciliation may be valuable.

## Introduction

Therapeutic interchange, or the substitution of a same-class drug for a pre-admission medication, theoretically allows healthcare systems to provide a safe yet cost-effective method to control pharmaceutical expenses and pharmacy size without compromising patient care.[1, 2] Narrow hospital formularies have been promoted as improving patient safety by enabling hospital clinicians and nurses to become familiar with a smaller set of medications; moreover, they reduce hospital costs. Hospitals that have implemented therapeutic interchange have reported savings ranging from less than $10,000 to greater than $1 million annually.[3] In 2010, fully 92% of hospitals reported using therapeutic interchange and a restricted hospital formulary.[4, 5] However, therapeutic interchange may also increase the risk of error by forcing a switch on admission from patients’ home medication to a different medication in the same class that is on the hospital formulary.[6] While benefits of hospital formularies have been explored, their associated risks are less well-established.

Critics argue that changing medications within a class may be less patient-centered, be less effective, cause more side effects, or promote a shift to more expensive medication use.[6–9] However, others argue that changes can generally be made safely, that outcomes are usually equivalent, and that a restricted formulary is necessary for efficiency and standardization of care.[1, 10–13] While such benefits may be substantive in the inpatient setting, there has been little research on the impact of therapeutic interchange on discharge medication reconciliation. At discharge, a medication reconciliation process should determine the patient’s new outpatient regimen, at which time either the original or the interchanged drug (or both, or neither) is selected, possibly leading to additional unintended medication discrepancies.[14, 15] Between one quarter to one half of discharge medication lists contain unexplained discrepancies.[15–17] The most common discrepancies are omitted medications (30–40% of errors), changes to dosage and/or frequency, duplication of prescriptions, and incomplete prescriptions.[18, 19] Whether therapeutic interchange contributes to this high rate of error at discharge is unknown.

Given the pervasiveness of therapeutic interchange in hospital settings and its potential contribution to post-discharge medication errors, we investigated the frequency and accuracy of therapeutic interchange during hospitalization, the extent to which interchanged medications are continued at discharge, and the association of therapeutic interchange with medication reconciliation errors at discharge in a single-center setting.

## Methods

### Study sample

We conducted a retrospective chart review of data collected from a previous study. The DIagnosing Systemic failures, Complexities and HARm in GEriatric discharges (DISCHARGE) study was a prospective, observational cohort study of patients 65 years or older admitted to Yale-New Haven Hospital for acute coronary syndrome, heart failure, or pneumonia between May 2009 and April 2010 who were subsequently discharged to home. Additional eligibility criteria included speaking English or Spanish, not being in hospice care, and participating in a telephone interview; caregivers could take part in lieu of patients. Patients were excluded if they appeared delirious or failed a mental status exam. The DISCHARGE study included an examination of medication reconciliation accuracy and patient understanding of medication changes post-discharge.[15] The study was approved by the Yale Human Investigation Committee and informed consent was obtained from all participants.

### Therapeutic interchange and medication reconciliation processes

During the time period of the study, the hospital used a combination of electronic health records (Sunrise Clinical Manager 5.8 (Eclipsys Corporation, now Allscripts, San Jose, CA)) and paper medical charts. The admitting physician recorded the admission medication list in a paper or electronic note. Medications prescribed during hospitalization were documented in handwritten or typed progress notes, medication orders were entered electronically by the providers, and medications dispensed were tracked by an electronic medication administration record (MAR). An attempt to order a non-formulary medication would generate a warning that the medication was not on formulary; however, no decision support or standardized automatic therapeutic interchange protocol was provided to the ordering provider to identify therapeutic equivalent doses of the formulary medication. The discharging physician used a prescription writer module to create the discharge medication list, which was integrated into the discharge instructions given to the patient. Clinicians performed the medication reconciliations manually as the electronic health record could not actively compare medication lists, and pharmacists did not systematically review the final discharge list.

We selected six drug classes for study of therapeutic interchange based on their frequency of therapeutic interchange, overall frequency of use, potential health impact in the event of medication error, and large variances in cost within each drug class:[3] proton pump inhibitors (PPIs), histamine H_2_-receptor antagonists (H2 blockers), hydroxymethylglutaryl CoA reductase inhibitors (statins), angiotensin-converting enzyme (ACE) inhibitors, angiotensin receptor blockers (ARBs), and inhaled corticosteroids (ICS). The formulary medications and their appropriate conversion dosages for the study institution during the study period are listed in S1 Table.

For the current study, we included patients who were taking at least one medication on admission that fell within the six drug classes listed above (“medications of interest”). We abstracted admission, inpatient, and discharge medication lists. For each patient, we tracked his/her medications of interest from admission to hospital stay to discharge. Using the admission medication list as the gold standard, we identified any therapeutic interchange (use of a formulary medication during hospitalization that was in the same class as a different home medication), change in dose or frequency, hold or discontinuation of medication, and addition of new medication that occurred between admission and discharge. A single reviewer examined the charts, seeking consensus with a second author for ambiguous cases.

According to the National Coordinating Council for Medication Error Reporting and Prevention, a medication error is “any preventable event that may cause or lead to inappropriate medication use or patient harm.”[20] We defined suspected medication errors as any medication discrepancies involving medications of interest that did not appear intentional (and thus may be inappropriate) based on review of the medical record, including progress notes, medication administration, medication orders, discharge summary, and discharge instructions to patients. Any mention of intention to change the medication (such as, “needs higher dose of statin”) was considered an intentional change even if it did not reference the medication or new dose explicitly. We did not collect data on intentional medication changes. A correct therapeutic interchange requires that the physician accurately converts the dose and frequency of the home medication to the equivalent formulary medication’s dose and frequency. We identified suspected medication conversion errors on admission as well as discrepancies in medication reconciliation at discharge. Any differences to the medications of interest between admission and discharge that were not intentional were classified as suspected medication reconciliation errors at discharge. We then examined whether therapeutic interchange at admission was associated with these suspected errors at discharge.

We further characterized the medications of interest by whether they were changed to a different drug (within the same drug class) at discharge compared to their home medication prior to admission and examined whether therapeutic interchange at admission was associated with these changes at discharge. A drug change is either intentional, meaning it was clinically indicated, or unintentional, meaning there was no apparent clinical indication and it is a suspected medication error.

The primary outcomes of this study included the percentage of medications of interest that were therapeutically interchanged during hospitalization and the frequency of suspected conversion errors made during the interchange, as well as the rate of suspected medication reconciliation errors at discharge associated with therapeutic interchange.

We captured demographic data about the patients from the electronic health record; these included age, sex, race/ethnicity and payor.

### Statistical analysis

We characterized the study sample, the frequency of therapeutic interchange, the frequency of medication conversion errors, and the frequency of medication reconciliation errors using descriptive statistics. We compared characteristics of patients in the interchange and non-interchange groups using chi squared tests or t-tests, as appropriate. We then calculated the relative risk of therapeutic interchange for medication reconciliation errors and the relative risk of therapeutic interchange for change of drug within the same class at the medication (not patient) level.

## Results

**Fig 1:**
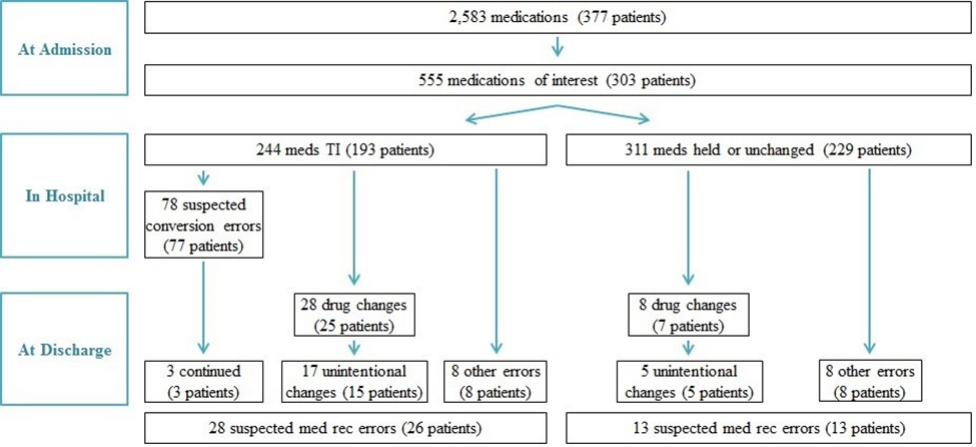
Flow diagram of study participants.

A total of 377 patients were enrolled in the DISCHARGE study, collectively taking 2,583 oral or inhaled medications on admission. Of these, 303 (80.4%) patients were taking 555 (21.5%) medications at admission that fell within the six drug classes of interest and were included in this analysis (Fig 1). The largest class of medications was statins, which accounted for 41% of the medications of interest. On average, 49% of these admission medications were non-formulary; however, rates for individual classes ranged from 20% for ACE inhibitors to 75% for PPIs (**Table 1**).

**Table 1:** Medications of interest characterized by drug class, formulary status and therapeutic interchange.

Admission Medication	Total	Non-formulary N (%)	Medication Status during Hospitalization		
			TI N (%)	Unchanged N (%)	Held N (%)
Angiotensin conversion enzyme inhibitor	122	25 (20%)	14 (11%)	77 (63%)	31 (25%)
Angiotensin receptor blocker	59	15 (25%)	11 (19%)	32 (54%)	16 (27%)
H2 blocker	25	14 (56%)	9 (36%)	12 (48%)	4 (16%)
Proton pump inhibitor	112	84 (75%)	59 (53%)	26 (23%)	27 (24%)
HMG-coA reductase inhibitor (“statin”)	227	129 (57%)	146 (64%)	69 (30%)	12 (5%)
Inhaled corticosteroid	10	6 (60%)	5 (50%)	4 (40%)	1 (10%)
Total	**555**	**273 (49%)**	**244 (44%)**	**220 (40%)**	**91 (16%)**

TI = therapeutic interchange

Of the 555 study medications, 244 (44.0%) were therapeutically interchanged to an approved formulary drug at admission, affecting 64% of the study patients who were taking at least one medication of interest. Characteristics of patients exposed to therapeutic interchange compared to those taking medications that were not interchanged are shown in **Table 2**; 119 patients fell into both cohorts because they were taking some medications that were and some medications that were not interchanged. There were no significant differences in characteristics between the two groups.

**Table 2:** Characteristics of patients in each cohort^a^.

Characteristic	Therapeutic interchange (N = 193)	No therapeutic interchange (N = 229)	P value
Mean age, years (SD)	76.3 (7.4)	77.6 (7.6)	0.07
Male sex, N (%)	108 (56.0)	129 (56.6)	0.90
Principal diagnosis, N (%)			
Acute coronary syndrome	112 (58.0)	121 (52.8)	0.29
Heart failure	76 (39.4)	97 (42.4)	0.54
Pneumonia	33 (17.1)	43 (18.8)	0.65
Race/ethnicity, N (%)			0.82
White	157 (81.4)	183 (80.3)	
Black	25 (13.0)	32 (14.0)	
Hispanic	8 (4.2)	9 (4.0)	
Payor, N (%)			0.39
Medicare	180 (93.3)	208 (91.2)	
Commercial	11 (5.7)	25 (8.8)	

^**a**^119 patients are present in both cohorts

Among the therapeutically interchanged drugs, we identified 78 (32.0%) suspected medication conversion errors, affecting 77 different patients; these medications were not converted to the equivalent dose and/or frequency of the formulary drugs (**Fig 1**). Most of the suspected conversion errors involved statins (79.2%). Of the 78 total suspected dose conversion errors made at admission, 3 (3.8%) were continued through discharge.

We identified suspected medication reconciliation errors at discharge involving 41 (7.4%) of the 555 medications of interest overall (**Fig 1**). Of these, 28 (68.3%) involved medications that had been therapeutically interchanged during admission. The discharge medication reconciliation error rate was 11.5% among the 244 therapeutically interchanged medications, compared with 4.2% among the 311 unchanged medications (relative risk [RR] 2.75, 95% confidence interval [CI] 1.45–5.19). The most common form of suspected medication reconciliation error at discharge involved unindicated substitution of a medication in the same drug class, with or without additional errors in dosage or frequency (first two rows in **Table 3**). Omission of a medication was also frequent; other suspected errors included duplication, inadvertent continuation, or incorrect dosing changes.

**Table 3:** Suspected medication reconciliation errors at discharge, characterized by type.

Type of Suspected Error at Discharge	TI (N)	No TI (N)
Different medication in same drug class as admission medication prescribed at discharge, equivalent in dose/frequency, without clear indication	10	2
Different medication in same drug class as admission medication prescribed at discharge, not equivalent in dose/frequency, without clear indication	5	3
Two medications in same drug class prescribed at discharge	3	1
Admission medication omitted at discharge without clear indication	6	4
Dose of admission medication incorrect at discharge	2	2
Admission medication continued at discharge when intended to be stopped	1	0
Admission medication prescribed at discharge when notes indicate plan to change to different medication	1	1
**Total**	**28**	**13**

TI = therapeutic interchange

Furthermore, a total of 36 (6.5%) of the 555 medications of interest were changed at discharge to a different medication within the same drug class as the patient’s original admission medication (Fig 1). The regimen change occurred in 28 of the 244 therapeutically interchanged medications (11.5%) as compared to 8 of the 311 non-therapeutically interchanged drugs (2.6%) (RR 4.46, 95% CI 2.07–9.61). Some of these drug changes were intentional consequences of inpatient management; for example, a patient admitted on esomeprazole was switched to a therapeutically equivalent dose of the formulary drug pantoprazole, but because of GI symptoms, the frequency of pantoprazole administration was increased, and she was discharged on this new regimen. However, for the majority of drug changes (17 of 28 therapeutically interchanged medications and 5 of 8 of those not therapeutically interchanged), the replacement appeared unintended and was classified not only as a drug change but also as a suspected medication reconciliation error at discharge.

## Discussion

In this single-center study, we found that, within six commonly used drug classes, nearly half of medications were therapeutically interchanged at admission, and one-third of these were likely converted incorrectly, subjecting hospitalized patients to inequivalent medication regimens. Furthermore, therapeutic interchange increased risk of suspected medication reconciliation errors by 2.75 times and increased risk of a change in medication within the same class at discharge by 4.5 times. Together, these results indicate that therapeutic interchange is a common process that affects patients’ medication regimens not only during hospitalization but beyond, and suggest that therapeutic interchange is a key contributor to medication reconciliation errors.

This study was limited to six classes of medications; however, of all eligible patients included in the original DISCHARGE study, 80.4% of patients were taking at least one medication in these six classes, making our study highly generalizable in this context. Over half of all eligible patients experienced at least one therapeutic interchange in just these six classes during hospitalization, stressing the importance of thorough evaluation of the effects that therapeutic interchange has on medication regimens and errors, as they may be wide-ranging. Previous studies characterizing medication errors made at time of hospital admission have not specifically examined errors related to therapeutic interchange.[21–24] Typically, such investigations focus on discrepancies made between the admission medication list and patients’ actual outpatient regimen when constructing the “best possible medication history,”[25] but do not explore therapeutic interchange as the next step of the transition process. Given the high rate of possible interchange errors, priority should also be given to avoiding interchange issues.

The high rate of suspected conversion errors at admission suggest that therapeutic equivalence was often not achieved during hospitalization. This creates an opportunity for adverse drug events and complications in inpatient care. Prior medication reconciliation studies, which have examined medication errors but have not explicitly discussed therapeutic interchange, have shown that between 27–40% of admission discrepancies either do cause harm or have the potential to cause harm.[21–23] Potential adverse events may be exacerbated by the fact that patients are often unaware of the changes in medication and do not have the means to verify their inpatient regimen for dosing accuracy, as illustrated in a study which found that 36% of patients subject to PPI therapeutic interchange during hospitalization were unaware that a substitution had occurred.[26]

Suspected conversion errors at admission also have potential for harm beyond hospitalization if they are not remedied at discharge.[21, 27] In this study, 3 of the 78 (3.8%) suspected conversion errors were continued at discharge, indicating that the patient may have continued the incorrect therapeutic interchange at home. At discharge, suspected medication reconciliation errors were identified in 7.4% of medications analyzed, affecting 12.5% of patients. This rate is comparable to other studies, although this study was more comprehensive than most by including errors of omission, duplication, changes in dosage and frequency, inadvertent continuations, and therapeutic interchange.[28] Again, while other types of medication errors at discharge have been detailed, discrepancies related to therapeutic interchange are rarely explicitly examined. Total suspected errors were almost three times more likely to occur in medications that were interchanged during hospitalization; many of these suspected errors involved medications that were not reverted back to the home regimen (first two rows of **Table 3)**, likely a consequence of therapeutic interchange. The potential for mistakes post-discharge is high as patients may take the incorrect dosage of a medication, take two medications of the same class, or discontinue a needed treatment.

Furthermore, we found that in 6.5% of cases, a patient’s admission medication changed to a different drug of the same class at discharge. Such changes in medication regimen, whether clinically indicated or not, affected 13% of patients who underwent at least one therapeutic interchange. This is in agreement with the 2014 findings of Glaholt *et al*. who observed that 15% of adult patients admitted to an academic medical center who experienced therapeutic interchange were not restarted on their original outpatient therapy.[14] In our study, medication switches were significantly more likely to occur to medications that were changed for formulary reasons than for medications that were unchanged or held during hospitalization.

Minimizing changes to drug regimens, especially in vulnerable populations such as the elderly in this study, is important in avoiding patient misunderstanding and potential adverse drug effects. Changes can be costly: patients may have a previous home supply available but be required to obtain a new prescription for a therapeutically equivalent drug following discharge. Subsequent confusion may increase the likelihood of medication duplication or omission, possibly putting the patient at risk for drug-drug interactions, side effects, and adverse reactions. For these reasons, providers should make changes from one medication to another carefully and only when indicated, and be aware of potential unintentional consequences. Of note, this study did not examine the potential consequences of suspected errors, nor did it analyze whether specific demographic factors contribute to therapeutic interchange errors.

Aside from improving provider awareness, we may be able to reduce errors related to therapeutic interchange by instituting systems-level changes to hospitals. For example, just as standardized protocols can improve medication reconciliation, the use of formalized tools could make therapeutic interchange safer. Medication reconciliation protocols should indicate which medications undergo therapeutic interchange at admission and include a means of ensuring that this interchange is reversed at discharge. Formulary conversion charts such as those in S1 Table should be readily accessible, embedded within the electronic health record, or provided through point-of-service clinical decision support to promote accurate changes. Another potential method of improvement is to include pharmacists in the medication reconciliation process. Not only do pharmacists have the training to assist in the process, but they are extremely familiar with their hospital’s formulary, which has been shown to specifically reduce therapeutic interchange errors.[24, 27, 29]

The use of an active electronic medication reconciliation system that can flag therapeutic interchanges may also aid providers.[30] The electronic health record used in this study included electronic medication lists but did not automatically compare admission to discharge lists. In 2013, 85% of hospitals had electronic health record systems that allowed at least basic medication reconciliation capability, though only 63% could electronically prescribe discharge medications.[31] These modules, though, may not yet be adequate for patient care. In one survey of 19 hospitals with electronic medication reconciliation capability, one third used a hybrid paper-electronic process because of dissatisfaction with the module.[32] By contrast, the capabilities of some systems allow for the computerized prompting of therapeutic interchange at admission with suggested dose conversions and can highlight medication changes during medication reconciliation at discharge. Ideally, evolving technology will reduce the failures observed in this study, including changes related to therapeutic interchange; however, at present, there is lack of implementation and standardization of these systems.

There are several limitations to this study. First, as a retrospective chart review, the admission medication list documented in the patient charts was treated as the gold standard although numerous studies have shown high rates of medication reconciliation errors at admission. Second, we could not verify intentional changes or suspected errors to regimens at admission or discharge with the patients’ actual providers; instead we relied on chart review including clinical documentation and medication orders and administration records. Thus, some of the suspected conversion errors on admission and suspected errors on discharge may in fact have been intentional. However, the 7.4% overall suspected error rate at discharge found in this study is comparable to other medication reconciliation studies, which range from a rate of 5% to 16%.[15–17] Third, the study is limited to patients from a single tertiary level hospital in an urban area and may not be generalizable to practices at other hospitals. Moreover, electronic health record capacity for medication reconciliation assistance is rapidly evolving, though there remains a wide range in complexity and effectiveness of these systems. Many hospitals today have more sophisticated systems than were available in this study. Fourth, limiting the investigation to six drug classes rather than all medications may affect overall rates of therapeutic interchange and suspected errors. However, as mentioned previously, these drug classes are among the most commonly used and commonly interchanged, and the suspected errors that occurred across all six classes suggest that similar errors may also be quite prevalent for other drug classes.

In this single-center study, we show that therapeutic interchange is prevalent in the hospital setting and elevates the risk for potential medication errors during and after hospitalization. While therapeutic interchange reduce inpatient pharmaceutical costs, its benefits should be carefully weighed against the potential for increased errors and adverse events and protocols should be specifically designed to reduce the risk of associated error.

## Supporting information

S1 Table Formulary conversions for drug classes of interest. (DOCX)

S1 File STROBE statement—Checklist of items that should be included in reports of *cross-sectional studies*. (DOC)

S2 File Deidentified study dataset. (XLSX)
