# Supplementary material for: Effect of therapeutic interchange on medication reconciliation during hospitalization and upon discharge in a geriatric population
Source: PLoS One. 2017 Oct 19;12(10):e0186075. doi: 10.1371/journal.pone.0186075 (PMC5648145; doi:10.1371/journal.pone.0186075)
Supplement: S1 Table — (DOCX) [file pone.0186075.s001.docx]

**S1 Table: Formulary Conversions for Drug Classes of Interest**

S1 Table 1a: Formulary Conversions for ACE Inhibitors

| FOR THE FOLLOWING ORDERS | | | | | | | | **INTERCHANGE USING** | |
| --- | --- | --- | --- | --- | --- | --- | --- | --- | --- |
| **benazepril** | **enalapril** | **fosinopril** | **moexipril** | **perindopril** | **ramipril** | **trandolapril** | **quinapril** | **captopril** | **lisinopril** |
| 5mg QD | ---- | 5mg QD | 3.75mg QD | 4mg QD | 1.25mg QD | 0.5mg QD | 5mg QD | 25mg QD | **5mg QD** |
| 10mg QD | 5mg BID | 10mg QD | 7.5mg QD | 8mg QD | 2.5mg QD | 1mg QD | 10mg QD | 25mg BID | **10mg QD** |
|  |  |  |  | 4mg BID |  |  |  |  |  |
| 20mg QD | 10mg BID* | 20mg QD | 15mg QD | ---- | 5 mg QD | 2mg QD | 20mg QD (HTN) | ---- | **20mg QD (HTN)** |
| 10mg BID |  |  |  |  |  |  | 20mg BID (CHF) |  | **20mg BID (CHF)** |
|  |  |  |  |  |  |  |  |  | ***20mg QD (HTN and CHF)** |
| 40mg QD | 20mg BID | 40mg QD | 30mg QD | ---- | 10mg QD | 4mg QD | 40mg QD | ---- | **40mg QD** |
| 20mg BID |  |  |  |  |  |  |  |  |  |
| 80mg QD | ---- | 80mg QD | ---- | ---- | 20mg QD | ---- | 80mg QD | ---- | **80mg QD** |
| 40mg BID |  |  |  |  |  |  |  |  |  |

QD = daily, BID = twice daily, HTN = hypertension, CHF = congestive heart failure

S1 Table 1b: Formulary Conversions for ARBs

| FOR THE FOLLOWING ORDERS | | | | | **INTERCHANGE USING** | |
| --- | --- | --- | --- | --- | --- | --- |
| **candesartan** | **eprosartan** | **irbesartan** | **olmesartan** | **telmisartan** | **losartan** | **valsartan** |
| 4mg QD | 400mg QD | 75mg QD | 5mg QD | ---- | **25mg QD** | **40mg QD** |
| 8mg QD | 600mg QD | 150mg QD | 20mg QD | 20mg QD | **50mg QD** | **80mg QD** |
| 16mg QD | 800mg QD | 300mg QD | 40mg QD | 40mg QD | **100mg QD** | **160mg QD** |
| 32mg QD |  |  |  | 80mg QD |  | **320mg QD** |

QD = daily, BID = twice daily

S1 Table 1c: Formulary Conversions for H2 Blockers

|  | FOR THE FOLLOWING ORDERS | | | **INTERCHANGE USING** |
| --- | --- | --- | --- | --- |
| **CRCL (ml/min)** | **cimetidine** | **nizatidine** | **ranitidine** | **famotidine** |
| <50 | <30 ml/min: 300mg BID | 150mg QD | 150mg QD | **20mg QD** |
|  | <10ml/min: 300mg QD | 150mg QOD |  |  |
| >50 | 300mg Q8H | 150mg BID 300mg QD | 150mg BID | **20mg BID** |
|  | 400mg BID |  |  |  |
|  | 800mg QD |  |  |  |

CRCL = creatinine clearance, QD = daily, QOD = every other day, BID = twice daily

S1 Table 1d: Formulary Conversions for PPIs

| FOR THE FOLLOWING ORDERS | | | | | **INTERCHANGE USING** |
| --- | --- | --- | --- | --- | --- |
| **esomeprazole** | **dexlansoprazole** | **lansoprazole** | **omeprazole** | **rabeprazole** | **pantoprazole** |
| 20mg QD | 30mg QD | 15mg QD | 20mg QD | 20mg QD | **40mg QD** |
| 40mg QD | 60mg QD | 30mg QD | 40mg QD |  |  |
|  |  |  | 20mg BID  40mg BID  60mg QD  60mg BID | 20mg BID | **40mg BID** |
| 40mg BID |  | 30mg BID |  | 40mg BID |  |
| 60mg QD | ---- | 60mg QD |  | 60mg QD |  |
| 60mg BID |  | 60mg BID |  | 60mg BID |  |
|  |  |  |  | 100mg QD |  |

QD = daily, BID = twice daily

S1 Table 1e: Formulary Conversions for Statins

| FOR THE FOLLOWING ORDERS | | | **INTERCHANGE USING*** | | |
| --- | --- | --- | --- | --- | --- |
| **fluvastatin** | **lovastatin** | **simvastatin** | **atorvastatin** | **rosuvastatin** | **pravastatin** |
| 20mg QD | 10mg QD | 5mg QD | **----** | **----** | **10mg QD** |
| 40mg QD | 20mg QD | 10mg QD | **----** | **----** | **20mg QD** |
| 80mg QD | 40mg QD | 20mg QD | **10mg QD** | **----** | **40mg QD** |
| ---- | 80mg QD | 40mg QD | **20mg QD** | **5mg QD** | **80mg QD** |
| ---- | ---- | 80mg QD | **40mg QD** | **10mg QD** | **----** |
| ---- | ---- | ---- | **80mg QD** | **20mg QD** | **----** |
|  |  |  |  | **40mg QD** |  |

QD = daily. *Pravastatin was on formulary throughout study period (May 2009 – Apr 2010).

Atorvastatin was on formulary from May 2009 – Oct 2009. Rosuvastatin was on formulary

from Aug 2009 – Apr 2010

S1 Table 1f: Formulary Conversions for inhaled corticosteroids

| FOR THE FOLLOWING ORDERS | | **INTERCHANGE USING** | |
| --- | --- | --- | --- |
| **budesonide** | **fluticasone** | **beclomethasone** | **mometasone** |
| 90mcg 1 puff BID  180mcg 1 puff BID | 44mcg 1 puff BID | **40mcg 1 puff BID 80mcg 1 puff BID** | **220mcg 1 puff QD** |
|  | 50mcg 1 puff BID |  |  |
|  | 100mcg 1 puff BID |  |  |
| 180mcg 2 puffs BID | 110mcg 2 puffs BID | **80mcg 2 puffs BID** | **220mcg 1 puff BID** |
|  | 220mcg 1 puff BID |  |  |
|  | 110mcg 4 puffs BID |  | **220mcg 2 puffs BID** |
|  | 220mcg 2 puffs BID |  |  |
|  | 250mcg 2 puffs BID |  |  |

QD = daily, BID = twice daily
